# Role of carbonate burial in Blue Carbon budgets

**DOI:** 10.1038/s41467-019-08842-6

**Published:** 2019-03-07

**Authors:** V. Saderne, N. R. Geraldi, P. I. Macreadie, D. T. Maher, J. J. Middelburg, O. Serrano, H. Almahasheer, A. Arias-Ortiz, M. Cusack, B. D. Eyre, J. W. Fourqurean, H. Kennedy, D. Krause-Jensen, T. Kuwae, P. S. Lavery, C. E. Lovelock, N. Marba, P. Masqué, M. A. Mateo, I. Mazarrasa, K. J. McGlathery, M. P. J. Oreska, C. J. Sanders, I. R. Santos, J. M. Smoak, T. Tanaya, K. Watanabe, C. M. Duarte

**Affiliations:** 1 King Abdullah University of Science and Technology (KAUST), Red Sea (RSRC) and Computational Bioscience (CBRC) Research Centers, Thuwal, 23955-6900 Saudi Arabia; 2 School of Life and Environmental Sciences, Centre for Integrative Ecology, Deakin University, Geelong, Victoria 3216 Australia; 3 Southern Cross Geoscience, Southern Cross University, Lismore, New South Wales 2480 Australia; 4 Department of Earth Sciences, Utrecht University, Vening Meineszgebouw A, Princetonlaan 8a, Utrecht, 3584 The Netherlands; 5 School of Science and Centre for Marine Ecosystems Research, Edith Cowan University, 270 Joondalup Drive, Joondalup, West Australia 6027 Australia; 6 Department of Biology, College of Science, Imam Abdulrahman Bin Faisal University (IAU), Dammam, 31441-1982 Saudi Arabia; 7 Institut de Ciència i Tecnologia Ambientals, Universitat Autònoma de Barcelona, Bellaterra, Barcelona, 08193 Spain; 8 Centre for Coastal Biogeochemistry, School of Environment, Science and Engineering, Southern Cross University, Lismore, New South Wales 2480 Australia; 9 Department of Biological Sciences, Center for Coastal oceans Research, Institute of Water and Environment, Florida International University, Miami 11200 SW 8th Street, Miami, Florida 33199 USA; 10 School of Ocean Sciences, Bangor University, Menai Bridge, Anglesey, Wales, LL59 5AB UK; 11 Department of Bioscience, Aarhus University, Vejlsøvej 25, Silkeborg, 8600 Denmark; 12 Arctic Research Centre, Department of Bioscience, Aarhus University, Ny Munkegade 114, Building 1540, Århus C, 8000 Denmark; 13 Coastal and Estuarine Environment Research Group, Port and Airport Research Institute, 3-1-1 Nagase, Yokosuka, 239-0826 Japan; 14 School of Biological Sciences, The University of Queensland, St Lucia, Brisbane, Queensland 4072 Australia; 15 Department of Global Change Research, IMEDEA (CSIC-UIB), Institut Mediterrani d’Estudis Avançats Miquel Marquès 21, Esporles (Illes Balears), 07190 Spain; 16 Oceans Institute and School of Physics, University of Western Australia, 35 Stirling Highway, Crawley, West Australia 6009 Australia; 17 Centro de Estudios Avanzados de Blanes, Consejo Superior de Investigaciones Cientificas, Blanes, 17300 Spain; 18 Environmental Hydraulics Institute “IH Cantabria”, C/Isabel Torres No 15, Parque Científico y Tecnológico de Cantabria, Universidad de Cantabria, Santander, 39011 Spain; 19 Department of Environmental Sciences, University of Virginia, Charlottesville, Virginia 22904 USA; 20 National Marine Science Centre, School of Environment, Science and Engineering, Southern Cross University, Cos Harbour, New South Wales 2450 Australia; 21 University of South Florida, St. Petersburg, Florida 33701 USA

## Abstract

Calcium carbonates (CaCO_3_) often accumulate in mangrove and seagrass sediments. As CaCO_3_ production emits CO_2_, there is concern that this may partially offset the role of Blue Carbon ecosystems as CO_2_ sinks through the burial of organic carbon (C_org_). A global collection of data on inorganic carbon burial rates (C_inorg_, 12% of CaCO_3_ mass) revealed global rates of 0.8 TgC_inorg_ yr^−1^ and 15–62 TgC_inorg_ yr^−1^ in mangrove and seagrass ecosystems, respectively. In seagrass, CaCO_3_ burial may correspond to an offset of 30% of the net CO_2_ sequestration. However, a mass balance assessment highlights that the C_inorg_ burial is mainly supported by inputs from adjacent ecosystems rather than by local calcification, and that Blue Carbon ecosystems are sites of net CaCO_3_ dissolution. Hence, CaCO_3_ burial in Blue Carbon ecosystems contribute to seabed elevation and therefore buffers sea-level rise, without undermining their role as CO_2_ sinks.

## Abstract (web summary)

Calcium carbonates (CaCO_3_) often accumulate in mangrove and seagrass sediments. Here the authors conducted a meta-analysis of inorganic carbon burial rates in mangrove and seagrass sediments and found that CaCO_3_ burial contributes to Blue Carbon ecosystems’ capacity to offset sea-level rise without undermining the role as CO_2_ sinks.

## Introduction

Mangrove forests and seagrass meadows have the capacity to elevate the seabed through the accretion of inorganic and organic particles^1^ at global rates of ~0.5 and ~0.2 cm yr^−1^, respectively^1^. Sediment accretion in mangrove forests and seagrass meadows leads to the sequestration of organic carbon (C_org_)^2,3^ originating from within and outside of the vegetated ecosystem^4^. Although mangroves and seagrass ecosystems occupy only a small fraction of the total coastal area (< 2%), they contribute 10% and 25% to the yearly C_org_ sequestration in the coastal zone^1,5^, respectively. Recognition of mangrove and seagrass meadows, together with saltmarshes, as sites of intense C_org_ burial led to the formulation of Blue Carbon strategies to mitigate and adapt to climate change, through conservation and restoration of these ecosystems^1,6–8^. The focus on Blue Carbon has provided substantial impetus to assess sediment C_org_ concentrations and burial rates in vegetated coastal ecosystems, which recently have been widely reviewed^9^.

C_org_ generally represents a minor fraction (2–3%) of buried material within mangrove and seagrass sediments^10,11^ (although this is highly variable^12^), the rest being siliciclastic and carbonate particles. A global assessment of the concentration of inorganic carbon concluded that C_inorg_ can exceed C_org_ concentration in seagrass sediments^13^. Seagrass and mangrove plants do not calcify per se; however, they provide habitats for an abundant associated calcifying fauna and flora (e.g., crabs, sea stars, snails, bivalves, calcified algae, foraminifera), whose shells and skeletons may be deposited and buried in the sediment along with the plant litter, and the organic and inorganic particles imported from adjacent ecosystems.

Counterintuitively, CaCO_3_ production represents a source of CO_2_ to the atmosphere, as calcification produces CO_2_ with a ratio of ~0.6 mol of CO_2_ emitted per mol of CaCO_3_ precipitated^14^. This has led to the argument that high CaCO_3_ burial may partially offset CO_2_ sequestration associated with C_org_ burial in some seagrass meadows and mangrove forests^15^. However, there are several caveats that affect these arguments and render inferences on the role of Blue Carbon ecosystems as net CO_2_ sinks or sources inconclusive^13,16^, based on the comparison of C_org_ and C_inorg_ sediment burial rates. To date, very few articles report the burial rates of CaCO_3_ in mangrove and seagrass ecosystems^15–17^, and the role of CaCO_3_ burial in sediments and CO_2_ emissions depends on the balance between dissolution and production. If CaCO_3_ dissolution equals local calcification, then the burial of CaCO_3_ is supported exclusively by allochthonous inputs and is neutral in terms of CO_2_ emissions or sequestration. If dissolution exceeds local calcification, then CaCO_3_ dynamics add to the CO_2_ sink capacity of Blue Carbon ecosystems, even if CaCO_3_, which must be subsidized from allochthonous sources, is buried in the sediments. Only if CaCO_3_ dissolution is lower than local calcification does CaCO_3_ burial result in CO_2_ emissions.

**Fig. 1:**
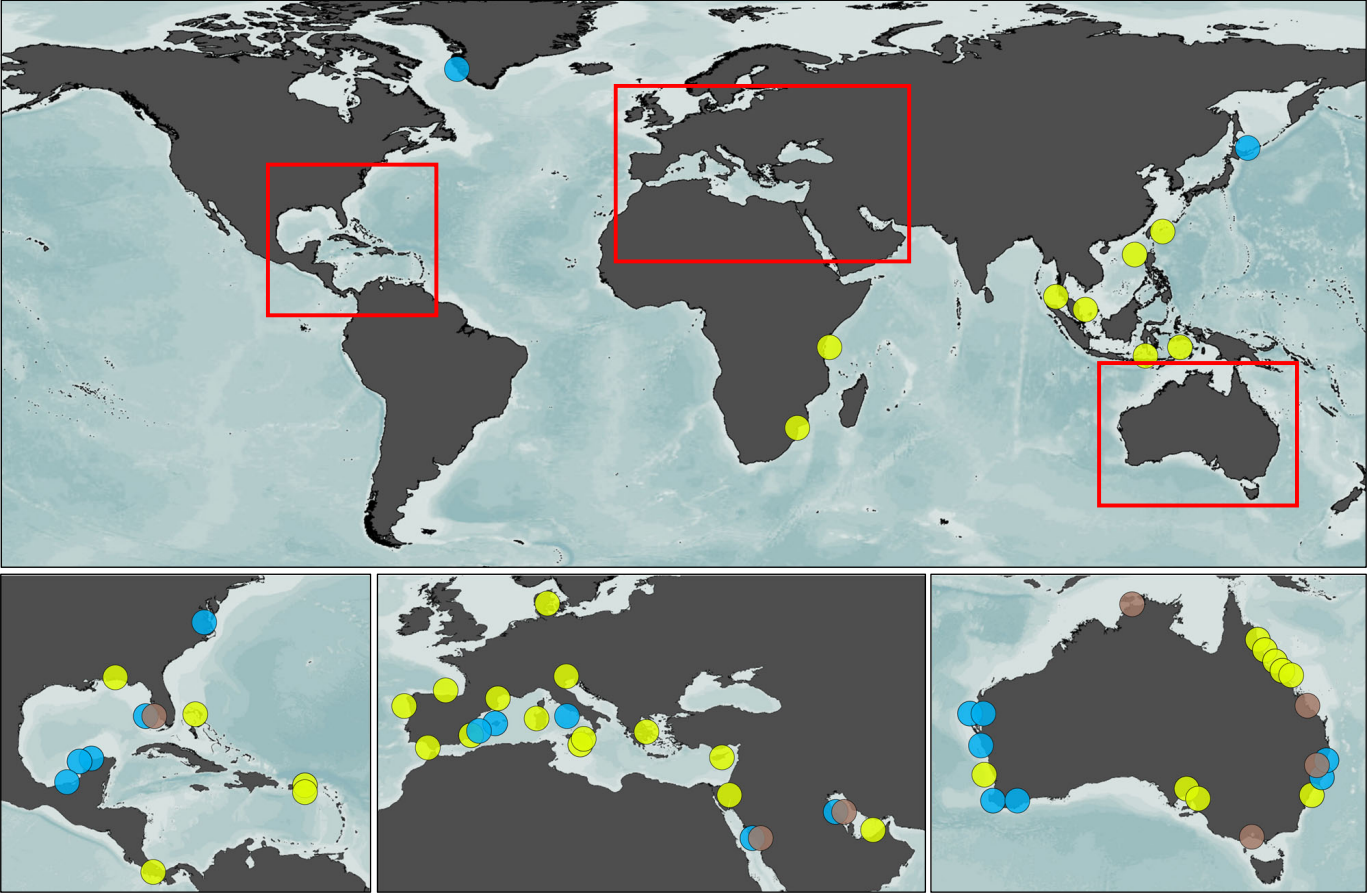
World map of sediment cores locations. Brown circles: mangrove cores locations; blue: seagrass cores locations; yellow: seagrass cores non-dated but with inorganic carbon content measured^13^

Here we address the current gap in global estimates of C_inorg_ burial in seagrass and mangrove ecosystems by providing first estimates of contemporary (last century) C_inorg_ burial rates. We rely on a compilation and analysis of data on sediment chronologies (i.e., including radiometric dating of sediment cores with ^210^Pb) and C_inorg_ concentrations from around the world (Fig. 1). We compare burial, calcification and dissolution rates in three locations where most of the carbon mass balance terms were available. We then address the role of CaCO_3_ burial in CO_2_ emissions by resolving the source of the CaCO_3_ buried in seagrass meadows as either allochthonous or autochthonous (i.e., from associated flora and fauna). We conclude that the high amounts of CaCO_3_ found in Blue Carbon sediments can not be converted into CO_2_ emissions.

## Results

### Global disparities in Blue Carbon sediments

CaCO_3_ supports an important part of sediment accretion rates (SARs) in seagrass ecosystems, although with large geographical disparities and a non-normal distribution (Shapiro–Wilks test, *p* < 0.001). Indeed, in 40% of global locations, the CaCO_3_ concentration was under 10% dry weight (DW), whereas in 28% of locations the CaCO_3_ content exceeded 80 %DW (see Supplementary Figure 1a). Overall, the median (interquartile range: IQR) global concentration of CaCO_3_ in seagrass meadow sediments was 61 (56) %DW (mean ± SE of 54 ± 7).

**Fig. 2:**
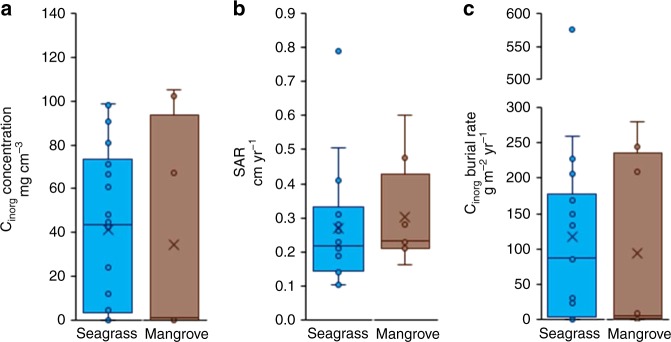
Sediment cores data. **a** Inorganic carbon (C_inorg_) concentration, **b** sediment accumulation rates (SAR), and **c** C_inorg_ burial rates. The x represents the mean. Bars are the first and last quartile

In mangrove forests, we observe a large difference between the mean (± SE) and the median (IQR) CaCO_3_ concentration: 21 ± 11% and 3 (31)%, respectively. This is explained by the strong non-normal distribution between the eight study locations examined, including a group of five locations with < 5 %DW CaCO_3_ in their sediments and three locations with CaCO_3_ contents between 20 and 75 %DW (Shapiro–Wilks test, *p* < 0.001, see Supplementary Fig. 1b). Converted into C_inorg_ concentrations (after normalization for the sediment bulk density), we obtain median (IQR) C_inorg_ concentrations in seagrass and mangrove sediments of 59 (66) and 1 (21) mgC_inorg_ cm^−3^, respectively (means ± SE of 63 ± 11 and 35 ± 17 mgC_inorg_ cm^−3^) (Fig. 2a).

**Fig. 3:**
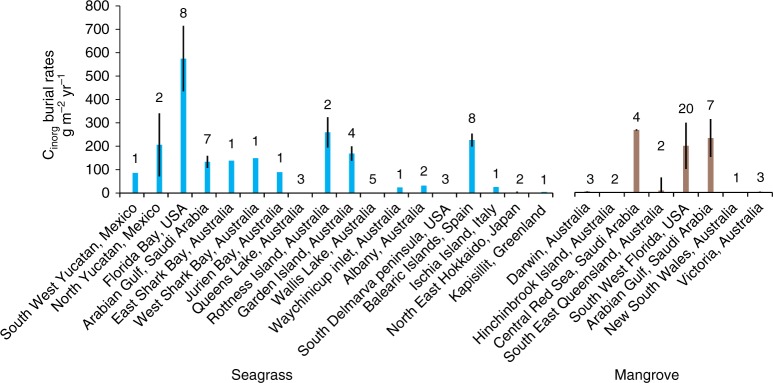
Inorganic carbon burial rates in all locations. Mean C_inorg_ burial rates in all locations in sediment cores for seagrass meadows and mangrove forests, organized from low to high latitudes. Bars are the SE. Labels are the number of cores per location

Using the median SARs in seagrass and mangrove ecosystems compiled in this study (0.22 and 0.23 cm yr^−1^, respectively; Fig. 2b), we estimate median (IQR) C_inorg_ burial rates in seagrass and mangrove ecosystems of 87 (154) and 6 (207) gC_inorg_ m^−2^ yr^−1^, respectively (means ± SE of 182 ± 94 and 90 ± 43 gC_inorg_ m^−2^ yr^−1^) (Fig. 2c, Fig. 3). These values correspond to vertical accretion rates of CaCO_3_ of the order of 0.1 and 0.001 cm yr^−1^ in seagrass and mangrove ecosystems, respectively. Our mean SAR values agree with previously reported global values^1,3^. However, our new estimates of burial rates are lower than the previous, indirect median estimate of C_inorg_ burial rate of 108 gC_inorg_ m^−2^ yr^−1^ (mean ± SE of 126 ± 31 gC_inorg_ m^−2^ yr^−1^) reported by Mazarrasa et al.^13^.

### Global annual burial rates of C_inorg_

Scaling up to the global seagrass coverage (150,000–600,000 km^2^)^9^, the annual burial rate of C_inorg_ ranged from 13 to 52 TgC_inorg_ yr^−1^ for the twentieth century (Table 1). Partitioning between tropical and non-tropical seagrass meadows as in Mazarrasa et al.^13^ showed that 90% of the global C_inorg_ burial occurs in the tropics (Table 1). In seagrass meadows, our estimates of global burial of C_inorg_ are equivalent to 31–55% of the available estimates of contemporary C_org_ burial rates (48–112 TgC_org_ yr^−1^)^1,7^, depending on the estimated global seagrass coverage considered. If all buried CaCO_3_ is locally produced (i.e., of autochthonous origin), the burial rates of C_inorg_ in seagrass meadows would represent emissions of 8–37 TgC yr^−1^ to the atmosphere and thus would offset their role as CO_2_ sinks through the sequestration of C_org_ by ~17–33%.

**Table 1:** Median (mean) global C_inorg_ burial rates for seagrass meadows and mangrove forests considering one, and, for seagrass, two world regions (tropical and higher latitudes)

		Burial rate, (TgC_inorg_ yr^−1^)			
		Global	Tropical	Higher lat.	Sum
Seagrass	This study	13(27)–52(109)	14(41)–57(163)	1(3)–5(14)	15(43)–62(177)
	Mazarassa et al.^13^	19(28)–65(79)			
Mangrove	This study	0.8(12)			

The extent of global mangrove coverage yields a median burial rate of 0.8 TgC_inorg_ yr^−1^ (mean of 13 TgC_inorg_ yr^−1^) (Table 1). This value should be considered as a first-order estimate because of the scarcity of data available on C_inorg_ burial rates in mangroves and because of the non-normal distribution between CaCO_3_-rich and CaCO_3_-poor mangrove sediments (Supplementary Figure 1b). When comparing with the global C_org_ burial rates estimate of 31 TgC_org_ yr^−1^ ^3^, the median C_inorg_ burial rates would correspond to a negligible reduction of net atmospheric CO_2_ uptake. However, assuming that all sedimentary CaCO_3_ was produced in situ, the C_inorg_ burial rates can largely outweigh C_org_ burial in CaCO_3_-rich mangroves. For example, the C_inorg_ burial rate corresponds to 10–20 times the C_org_ burial rate in the Arabian Peninsula^17–19^.

## Discussion

CaCO_3_ burial in Blue Carbon ecosystems is the balance between inputs (autochthonous and allochthonous) and losses (dissolution and export). Assessments of the mass balance of CaCO_3_ in seagrass meadows are few and none have been reported, to our knowledge, in mangrove forests. For seagrass ecosystems, we assessed the balance between calcification, dissolution and burial of CaCO_3_ in three locations: the Balearic Islands, Spain^20,21^, Shark Bay in Western Australia^22^ and Florida Bay, USA^23,24^ (Table 2).

**Table 2:** Burial rates of CaCO_3_ compared to calcification rates in seagrass ecosystems

	Community production rate of CaCO_3_		Community net calcification rate		Sediment		
	gCaCO_3_ m^−2^ yr^−1^	gC_inorg_ m^−2^ yr^−1^	gCaCO_3_ m^−2^ yr^−1^	gC_inorg_ m^−2^ yr^−1^	%CaCO_3_	gCaCO_3_ m^−2^ yr^−1^	gC_inorg_ m^−2^ yr^−1^
Florida Bay, USA	626^23,24^	75	18^25^	2.2	82 ± 2	4792 ± 756	756 ± 91
Balearic Islands, Spain	68^20^	8	51^21^	6	81 ± 3	1886 ± 214	226 ± 30
West Shark Bay, Australia	375 ± 62^22^	45 ± 7	295^22^	35	60 ± 5	1240 ± 232	149 ± 30

Comparison between seagrass-associated community production rate of carbonate (obtained from standing stock assessments and leaves or calcifiers turnover rates) and community net calcification rates (balance between calcification and dissolution, calculated from variations of total alkalinity) from the literature, and carbonate burial rate in three locations with carbonate-rich sediments

The most comprehensive assessment of seagrass carbon budgets is that reported for a Mediterranean *Posidonia oceanica* meadow at Magalluf (Mallorca Island, Spain)^20,21,25,26^. In this meadow, Barrón et al.^21^ estimated a net CO_2_ uptake by primary production of 8.4 gC m^−2^ yr^−1^. This estimate was corroborated by the C_org_ burial rate, estimated independently, at 9 ± 2 gC_org_ m^−2^ yr^−1^ ^27^. Barrón et al.^21^ also estimated net calcification rates of 51 gCaCO_3_ m^−2^ yr^−1^, corresponding to 6 gC_inorg_ m^−2^ yr^−1^. This amount of calcification would result in a CO_2_ emission of 3.6 gC m^−2^ yr^−1^ (0.6-fold the net calcification^14^). The CO_2_ emission by calcification therefore represents an offset of 40% of the CO_2_ uptake from net primary production (thereby yielding a total CO_2_ sequestration of 4.8 gC m^−2^ yr^−1^ ^21^). However, the C_inorg_ burial rate in this meadow is estimated here at 226 gC_inorg_ m^−2^ yr^−1^. This is two orders of magnitude greater than the net calcification rate of 6 gC_inorg_ m^−2^ yr^−1^ ^21^(Table 2). This implies that about 90% of the CaCO_3_ burial in this seagrass meadow must be supported by allochthonous inputs. Therefore, calculation of the CO_2_ sequestration by comparing C_org_ and C_inorg_ burial rates or stocks would have concluded that this meadow is a strong source of CO_2_, whereas estimates of calcification rates and net primary production concludes that it is a sink (as confirmed independently through air–sea flux measurements^26^).

Similarly, in Shark Bay, the burial of C_inorg_ is four times higher than the independently reported net calcification rate^22^ (Table 2). This again could require large allochthonous carbonate inputs.

In Florida Bay, the low ratio between C_org_ and C_inorg_ concentration in the sediment (g cm^−3^) implied that seagrass meadows may be a net source of CO_2_ to the atmosphere^15^. However, such assessment requires consideration of the full carbon mass balance, including accounting for allochthonous inorganic carbon inputs and the balance between calcification and dissolution in the meadows. The contemporary C_inorg_ burial rates in Florida Bay are approximately ninefold higher than the global median, whereas median SAR is about fourfold higher than estimated globally, in an area where 80% of the sediment dry mass is composed of CaCO_3_. However, attempts to assess the gross or net calcification rates in the area yielded values one and two orders of magnitude lower than the estimated CaCO_3_ burial rates (Table 2)^23,24^. In contrast, past geological work in the Bay has suggested that it is a net producer of CaCO_3_^28^. It is likely to be that some areas within this large Bay act as sources of CaCO_3_ and some others as sinks, helping explain the discrepancy between reported production and burial estimates. Hence, internal redistribution of CaCO_3_ production within Florida Bay needs to be considered when drawing inferences on the role of seagrass meadows from sediment composition.

These three example locations are in areas close to coral reefs and/or terrestrial lithogenic sources of CaCO_3_. We could not find estimates of calcification rates (net or gross) in areas without external sources of CaCO_3_. The discrepancies between calcification rates and burial rates in the three example locations could indicate that an important fraction of CaCO_3_ burial (> 90%) is supported by CaCO_3_ produced elsewhere and trapped in the seagrass sediments. This conclusion is consistent with comparable C_inorg_ concentrations within and outside seagrass meadows, whereas, in contrast, C_org_ concentrations are higher in seagrass sediments^13^. A large role of C_inorg_ import is also consistent with the large CaCO_3_ export from coral reefs to adjacent waters, equivalent to 25–50% of the CaCO_3_ production, predominantly to reef lagoons^27^, where seagrass meadows and mangroves often grow. Mangroves, seagrass and saltmarsh ecosystems are likely to be sites of net carbonate dissolution. Roots of marine plants release organic compounds and oxygenate the sediments during the day, promoting microbial aerobic remineralization of organic matter, thereby increasing sedimentary respiratory CO_2_^29,30^ and/or stimulating the re-oxidation of reduced metabolites. These processes result in the release of strong acids (e.g., H_2_SO_4_, HNO_3_)^31–33^, which leads to CaCO_3_ dissolution in the sediment^34,35^ (although re-precipitation can occur^34^).

Dissolution of CaCO_3_ might also be influenced by the CO_2_ system in the water column of Blue Carbon ecosystems. Respiration and photosynthesis of the flora and fauna, together with sediment redox processes in seagrass and mangrove ecosystems, strongly influence the chemistry of the water column, generating large diel amplitudes of the saturation state for CaCO_3_ (Ω) with a tendency towards dissolution or the reduction of calcification at nighttime, amplified at low tide^36–40^. The dissolution of allochthonous CaCO_3_ in carbonate platform areas, caused directly or indirectly by metabolism of the marine vegetation and associated biota, leads to a reduction in pCO_2_ through the release of fossilized total alkalinity. This sink of atmospheric CO_2_ should be incorporated into the Blue Carbon framework. A recent assessment considers alkalinity addition through the dissolution of allochthonous carbonate as a very effective geo-engineering approach to remove atmospheric CO_2_ and mitigate climate change^41,42^.

Similarly, saltmarshes are not known to host high levels of calcifying organisms but can accumulate CaCO_3_ from allochthonous sources. In arid tropical saltmarshes of the Western Arabian Gulf, dominated by succulent shrubs, a concentration of CaCO_3_ of 57 ± 8% in sediments and a contemporary burial rate of C_inorg_ of 100 ± 15 gC_inorg_ m^−2^ yr^−1^ (mean ± SE) were found^17^. In a temperate saltmarsh of the Western Scheldt estuary in the Netherlands, a concentration of CaCO_3_ of 14 ± 1% and a high contemporary burial rate of 467 ± 99 gC_inorg_ m^−2^ yr^−1^, mostly due to high SAR (1.1 ± 0.3 cm yr^−1^), were measured^43^. Yet, this does not imply that CaCO_3_ dynamics have a negligible role in saltmarsh carbon budgets, as they may still act as sites of net dissolution of CaCO_3_, adding to the removal of CO_2_ associated with C_org_ burial. A dissolution rate of 24–96 gC_inorg_ m^−2^ yr^−1^ was estimated in the sediment of the saltmarshes of the Eastern Scheldt estuary, corresponding to ~85% of the C_inorg_ burial rate^44^.

**Fig. 4:**
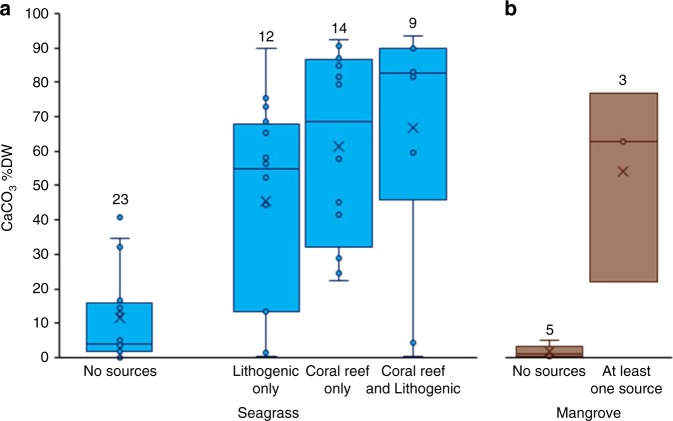
Allochthonous sources and inorganic carbon in sediments. **a** %DW of carbonates (CaCO_3_) in seagrass sediments depending on the presence of potential allochthonous sources (lithogenic and/or coral reefs). All data distributions for locations with allochthonous sources are significantly different to the distributions for locations without allochthonous sources (Mann–Whitney *U-*tests, all *p* < 0.001). **b** %DW CaCO_3_ in mangrove sediments with and without allochthonous sources (coral reef and lithogenic source). Number on top of the box plots indicate the number of locations. The *x* represents the mean. Bars are the first and last quartile

To further examine the conclusion that Blue Carbon ecosystems are sites of substantial allochthonous CaCO_3_ deposition, based on existing mass balances for Blue Carbon sediments, we examined (qualitatively) the relationship between the CaCO_3_ %DW in sediments and the presence/absence of sources of CaCO_3_ adjacent to the coring locations, including coral reefs and terrestrial lithogenic sources of CaCO_3_ (Fig. 4, see dataset in Supplementary Information). Seagrass and mangrove ecosystems without potentially large adjacent allochthonous CaCO_3_ sources have a remarkably lower median (IQR) sediment CaCO_3_ content of 4 (15) and 1 (1) %DW (means ± SE of 11 ± 4 and 1.7 ± 0.8 %DW), respectively, compared with59 (51) and 61 (27) %DW (means ± SE of 56 ± 5 and 53 ± 16 %DW) when at least one allochthonous CaCO_3_ source was present (Fig. 3). For sediments in seagrass meadows, the presence of coral reefs (*t*-value = 4.68, df = 48.5, *p* < 0.0001) and lithogenic sources (*t*-value = 4.76, df = 57.3, *p* < 0.0001) increased the amount of CaCO_3_ in the sediment. However, there was a significant interaction between these factors (*t*-value = − 3.29, df = 53.2, *p* = 0.0018), because the CaCO_3_ %DW in the presence of both allochthonous sources was less than would be expected if these variables were additive. The presence/absence of coral reefs and lithogenic sources accounted for 36% of the variation in CaCO_3_, whereas the random variables (study, lithology grouping and marine province) accounted for 54% of the variation in CaCO_3_ (see Methods for model description). Mangrove sediment samples showed a similar pattern to the seagrass meadows and the presence of allochthonous sources had a marginally significant positive effect on the amount of CaCO_3_ in the sediment (*t*-value = 4.29, df = 1.81, *p* = 0.0596). The presence/absence of a CaCO_3_ source accounted for 71% of the variation of in CaCO_3_ within mangrove sediments, whereas the random variables accounted for 20% of the variation in CaCO_3_. In testing for biases of outlying cores and studies, we found that one study from Western Florida had an outlying data point that disproportionality skewed the results. The study from Western Florida had relatively low CaCO_3_ but did have an allochthonous source of CaCO_3_. When this study was removed from the analysis, the presence of an allochthonous source became significant (*t*-value = 7.92, df = 4.16, *p* = 0.0012). This highlights the need for more studies in mangrove sediments to determine the global influence of allochthonous sources on CaCO_3_ content.

In seagrass meadows, the median (IQR) C_inorg_ burial rate found in areas where no allochthonous sources were identified was 1 (13) gC_inorg_ m^−2^ yr^−1^ (mean ± SE of 8 ± 4), only 1.1% of the global median. This contrast is consistent with our hypothesis that much of the C_inorg_ buried in seagrass and mangrove sediments is allochthonous. It explains the non-normal distribution of CaCO_3_ concentrations observed in sediments of seagrass and mangroves (Supplementary Figure 1), and indicates that the import of CaCO_3_ from carbonate-forming ecosystems or adjacent karstic areas is the norm^27^. The global burial rate of C_inorg_ in seagrass meadows is between a third to a half of their C_org_ burial rate^1,7^, whereas for mangroves our first estimate of global C_inorg_ burial rates is only 3% of the C_org_ burial rate^3^. If the buried CaCO_3_ and C_org_ in seagrass sediments were produced entirely in situ, C_inorg_ burial would offset up to a third of CO_2_ sequestration through C_org_ burial, particularly in tropical seagrass ecosystems where ~90% of the global C_inorg_ burial occurs. However, imbalances between production, dissolution and burial, and the observation of much higher CaCO_3_ concentrations in sediments near lithogenic formations and coral reefs, suggest that, where present, allochthonous CaCO_3_ inputs are substantial and support most of the net CaCO_3_ burial.

Locally, despite supporting significant CaCO_3_ burial, Blue Carbon ecosystems may be sites where imported CaCO_3_ dissolves, strengthening rather than weakening the capacity of these ecosystems to sequester CO_2_. Whereas there is emphasis on apportioning the sources of autochthonous and allochthonous C_org_ in Blue Carbon sediments (up to 50% of the buried C_org_)^4,9^, determining the sources of CaCO_3_ in Blue Carbon sediments is just as important, to resolve the role of vegetated coastal ecosystems as CO_2_ sinks and, hence, their potential to support climate change mitigation. The current focus on C_org_ budgets in vegetated coastal ecosystems needs to be augmented with integrative assessments of organic and inorganic carbon fluxes and budgets, including both allochthonous and autochthonous inputs. Moreover, these assessments must consider the sources and fate of carbon exchanged between Blue Carbon and adjacent ecosystems, as Blue Carbon ecosystems export important amounts of their organic production^45–47^ but also import significant amounts of CaCO_3_ and organic matter from adjacent sources. A comparison of paired vegetated and unvegetated sediment CaCO_3_ %DW showed that vegetated and adjacent unvegetated sediments have similar carbonate concentrations, both using standard parametric statistics (general linear model (GLM), *t*-value = 1.32, df = 83.1, *p* *=* 0.191) and meta-analysis (*z*-value = 0.88, *p* = 0.379; Supplementary Fig. 2A,B), which also showed no evidence for reporting bias (all points within the 95% confidence lines of the funnel plot, Supplementary Fig. 2C. This provides further support to the hypothesis that much of the carbonate buried in vegetated coastal sediments derives from allochthonous sources rather than being produced within the habitat.

Inorganic carbon burial in Blue Carbon ecosystems has been overlooked, with the rates compiled here representing the first direct estimates reported in the literature. These estimates confirm that seagrass ecosystems, and to lesser extent mangrove ecosystems, are intense sites of CaCO_3_ burial, supporting sediment accretion. CaCO_3_ burial is a fundamental process supporting the role of Blue Carbon ecosystems in climate change adaptation, which is underpinned by their capacity to rapidly accrete sediments, reducing relative SLR by raising the seafloor^1,17^.

## Methods

### Calculation of the C_inorg_ accretion rate

We searched the peer-reviewed literature for data on sediment cores dated with ^210^Pb, including CaCO_3_ or C_inorg_ concentration in seagrass and mangrove sediments. Search terms on Google Scholar were seagrass OR mangrove AND 210Pb OR SAR OR sediment accretion rate. We then searched returned articles that contained data on SAR and CaCO_3_ or C_inorg_ data. We found only one study presenting CaCO_3_ content in a dated sediment core. However, we found 15 and 22 studies with SAR for seagrass and mangrove sediments, respectively. To obtain the CaCO_3_ or C_inorg_ concentrations needed to calculate C_inorg_ burial rates, we used the database of Mazarrasa et al. ^13^, which was the most recent exhaustive compilation of sediment cores from Blue Carbon habitats, for data on CaCO_3_ in seagrass sediments. We also contacted experts in Blue Carbon studies (published studies using cores from Blue Carbon habitats) for unpublished CaCO_3_ sediment concentration data (see data and references in Supplementary Data 1). In total, we compiled 42 and 53 ^210^Pb dated cores with CaCO_3_ content in mangrove and seagrass ecosystems, respectively (see PRISMA checklist and flow diagram^48^ in Supplementary Note 1).

The SARs (cm yr^−1^) from the literature were re-calculated according to the constant flux–constant sedimentation model^49^, to have a coherent and comparable dating system between all cores. The CaCO_3_ concentration (% sediment DW) was calculated as the mean between all slices younger than 1900, for cores with the contemporary ^210^Pb chronologies. The C_inorg_ concentration in sediment (gC_inorg_ m^−3^) was calculated from the dry bulk density (g m^−3^) and the percentage of CaCO_3_ content (using sediment DW), considering a mass ratio of 12% carbon in CaCO_3_. The C_inorg_ burial rate (gC_inorg_ m^−2^ yr^−1^) was then calculated as the product of the SAR and the C_inorg_ concentration for each sediment core. Cores with negligible content of CaCO_3_ were also included in the calculation (see Supplementary Figure 1).

All cores from the same site or area and with similar presence or absence of allochthonous sources of CaCO_3_ (see below) were treated as replicates for a global location and averaged for the analysis (geologic grouping). For seagrass, the 51 cores dated with ^210^Pb were grouped into 17 locations (Figs. 2, 3). For mangroves, we compiled a total of 42 cores dated with ^210^Pb in 8 locations (Figs. 2, 3). Seagrass locations ranged from tropical to sub-arctic locations, with 50% of estimates derived from tropical and subtropical locations and 50% from higher latitudes. Mangrove sediment derived mostly from subtropical locations (seven out of eight locations), particularly in Australia and the Arabian Peninsula (Supplementary Figure 2).

### Determination of the influence of allochthonous sources of CaCO_3_

We analysed the influence of the presence/absence of proximity of coral reefs and continental surface lithology (qualitative data), as potential allochthonous sources of CaCO_3_ in seagrass and mangrove sediments (in %DW) (see dataset in Supplementary Data 1). For seagrass, we expanded our dataset by including CaCO_3_ concentrations from 264 cores compiled by Mazarrasa et al.^13^, reaching a total of 341 cores with measured CaCO_3_ %DW.

We estimated the presence/absence of coral reefs using the map of the global distribution of warm-water coral reefs compiled by the UNEP-WCMC^50^ and the presence/absence of nearby lithogenic sources using the global lithology map of Hartmann and Moosdorf^51^ and the world soil map of the FAO/UNESCO (http://www.fao.org/soils-portal/en/). The coring locations were associated with climate regions following the Köppen–Geiger classification system^52^.

### Statistical analysis

All data distributions were tested for normality to determine the most reliable central tendency measured with Shapiro–Wilks normality test (Statistica, Dell Software). None of the datasets of SAR, C_inorg_ concentration, C_inorg_ burial rate or CaCO_3_ %DW were normally distributed (all *p* < 0.05). We therefore chose to use the median (IQR) as the most appropriate description of central tendency. Traditional meta-analysis tools, which calculate effect sizes to standardize the difference between control and experimental treatments, thereby allowing comparison among disparate response variables and weighting to account for unequal variance among studies, could not be used for this analysis for multiple reasons. These reasons include that the question posed and the studies available did not include experimental designs with paired control and experimental plots required for effect size calculations, that there was a single response variable facilitating direct comparison and data integration, and, most importantly, that we used the raw data for each core. Instead, we ran a statistical test using a mixed effect GLM to determine the effect of coral reefs and lithogenic sources on the CaCO_3_ %DW of the sediment. For sediments within seagrass meadows, the GLM included two fixed factors (presence/absence of coral reefs and of lithogenic sources), as well as the interaction between the two factors. For sediment within mangrove forests, the GLM included one fixed factor (presence/absence of allochthonous sources), because replication did not exist for all combinations of the two factors. The data had unequal samples among studies and studies were not evenly distributed around the globe (Fig. 1), which could result in pseudo-replication and biased results. To account for the data structure and minimize non-independence, we included three separate random variables, which included study, lithology grouping and marine province. The marine province was determined for each sample location using the marine provinces of the world as defined by Spalding et al.^53^. Separate models where run for seagrass and mangrove sites. The statistical model was produced using the lmer function within the lme4 package^54^ and *p*-values were calculated with the lmerTest package^55^. The *R*^2^ was calculated for the fixed and random effects using the r.squared GLMM function in the MuMIn package^56^. The response variable was log transformed, which improved the model fit compared with raw data. The model fit was assessed by plotting the *Q*–*Q* plot (linear relationship) and the fitted values compared with the residuals (random distribution). To test whether individual cores or studies were biased and having a disproportionate influence on findings, we systematically removed any studies that contained outlying samples as determined from being outside the 95% confidence interval for the fitted values vs. residuals comparison using the plot model function from sjPlot package^57^. This analysis was conducted in R version 3.4.2.

Reporting bias and its effect on findings is an important consideration for meta-analyses^58^ and when the result from a meta-analysis is not the same as it would have been if data from all correctly conducted studies were included in the analysis^59^. A main cause of reporting bias is not publishing research because of a lack of merit as determined by the researcher, reviewer or editor^60^. As indicated by the data inclusion flow diagram (Supplementary Fig. 3), researchers often measured but did not publish data on soil CaCO_3_ content and authors needed to be directly contacted for these results. In addition, the researchers not only provided information from published studies but also unpublished data on CaCO_3_ content (10 of 51 seagrass studies included in the analysis were not published). For these reasons, it is unlikely to be that our findings were affected by reporting bias. A subset of data collected for this study included the appropriate information to run both a GLM and a traditional meta-analysis (effect size could be calculated between paired data). The data included information from nine studies that measured the CaCO_3_ content of sediment from both vegetated and unvegetated habitats. There were 92 core samples with 32 from unvegetated and 60 from vegetated habitats (Supplementary Fig. 2A). The GLM followed the same procedures as detailed in the main text, except it had only two random factors, study and marine province, because study and lithology grouping differed in only one instance. For the meta-analysis, the data were paired for each study and the mean CaCO_3_ %DW, number of samples and SD were calculated for vegetated and unvegetated cores for each study. Two studies included in the GLM were removed for the meta-analysis, because they only had one core for an unvegetated habitat and SD could not be calculated, leaving seven comparisons for this analysis. Hedges’ *g* was calculated for the effect size following Borenstein et al.^60^ (Eqs. 1–3) and a variance for each effect size was also calculated (*V*_*g*_)^59^ (Eq. 4), as indicated by equations:

1 $${\mathrm{Hedges}}'\, {g} = \frac{{\left( {X_{\mathrm{E}} - X_{\mathrm{C}}} \right)J}}{{\mathrm{SD}}_{\mathrm{pooled}}}$$

2 $${{\mathrm{SD}}_{\mathrm{pooled}}} = \sqrt {\frac{{\left( {n_{\mathrm{E}}-1} \right)\left( {{\mathrm{SD}}_{\mathrm{E}}} \right)^{2} + \left( {n_{\mathrm{C}}-1} \right)\left( {{\mathrm{SD}}_{\mathrm{C}}} \right)^{2}}}{{n_{\mathrm{E}} + n_{\mathrm{C}} - 2}}}$$

3 $$J = 1 - \frac{3}{{4\left( {n_{\mathrm{E}} + n_{\mathrm{C}} - 2} \right) - 1}}$$

4 $$V_{g} = \frac{{n_{\mathrm{E}} + n_{\mathrm{C}}}}{{n_{\mathrm{E}} \times n_{\mathrm{C}}}} + \frac{{g^{2}}}{{2\left( {n_{\mathrm{E}}+ n_{\mathrm{C}}} \right)}}$$

*X*_E_ and *X*_C_ are the mean (*n* is sample size) of vegetated and unvegetated sediments, along with SD_pooled_ and *J*, which accounts for biases associated with different sample sizes. The meta-analysis included the same two random variables as the GLM and was conducted using the rma.mv function from the metafor package^61^.

### Calculation of global yearly burial rates of C_inorg_

The global annual burial of inorganic carbon (TgC_inorg_ yr^−1^) in seagrass meadows was calculated as the product of the global median C_inorg_ burial rates and the estimated global seagrass area, which ranges from 150,000 to 600,000 km^2^^9^. We also calculated the global annual burial of C_inorg_ as the sum of separate calculations for tropical and arid climates and meadows at higher latitude climates. Median C_inorg_ burial rates were calculated for tropical (core locations with tropical and hot desert climates) and non-tropical areas (temperate, continental and polar climates) and multiplied by the global seagrass cover range under the assumption that 2/3 of the seagrass area is in the tropical and subtropical zone^13^. The global annual burial of inorganic carbon (TgC_inorg_ yr^−1^) in mangroves was calculated as the product of the global median C_inorg_ burial rates and the estimated global mangrove cover of 137,760 km^2^^62^.

## Supplementary information

Supplementary Information

Peer Review File

Description of Additional Supplementary Files

Supplementary Data 1

## Data Availability

The dataset is available as Supplementary Data 1.
