# Supplementary material for: Role of carbonate burial in Blue Carbon budgets
Source: Nat Commun. 2019 Mar 7;10:1106. doi: 10.1038/s41467-019-08842-6 (PMC6405941; doi:10.1038/s41467-019-08842-6)
Supplement: Supplementary file 1 — Supplementary Information [file 41467_2019_8842_MOESM1_ESM.pdf]

Role of carbonate burial in Blue Carbon budgets

Saderne et al., 2019

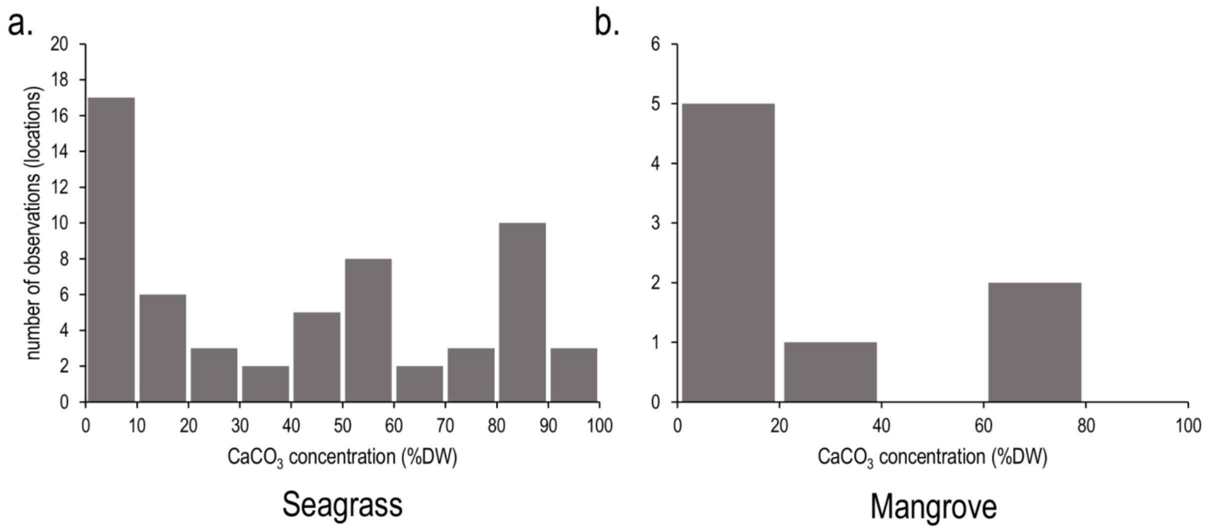

Supplementary Figure.1. Distribution of $CaCO_3$ content (%DW) in (a) seagrass and (b) mangrove locations.

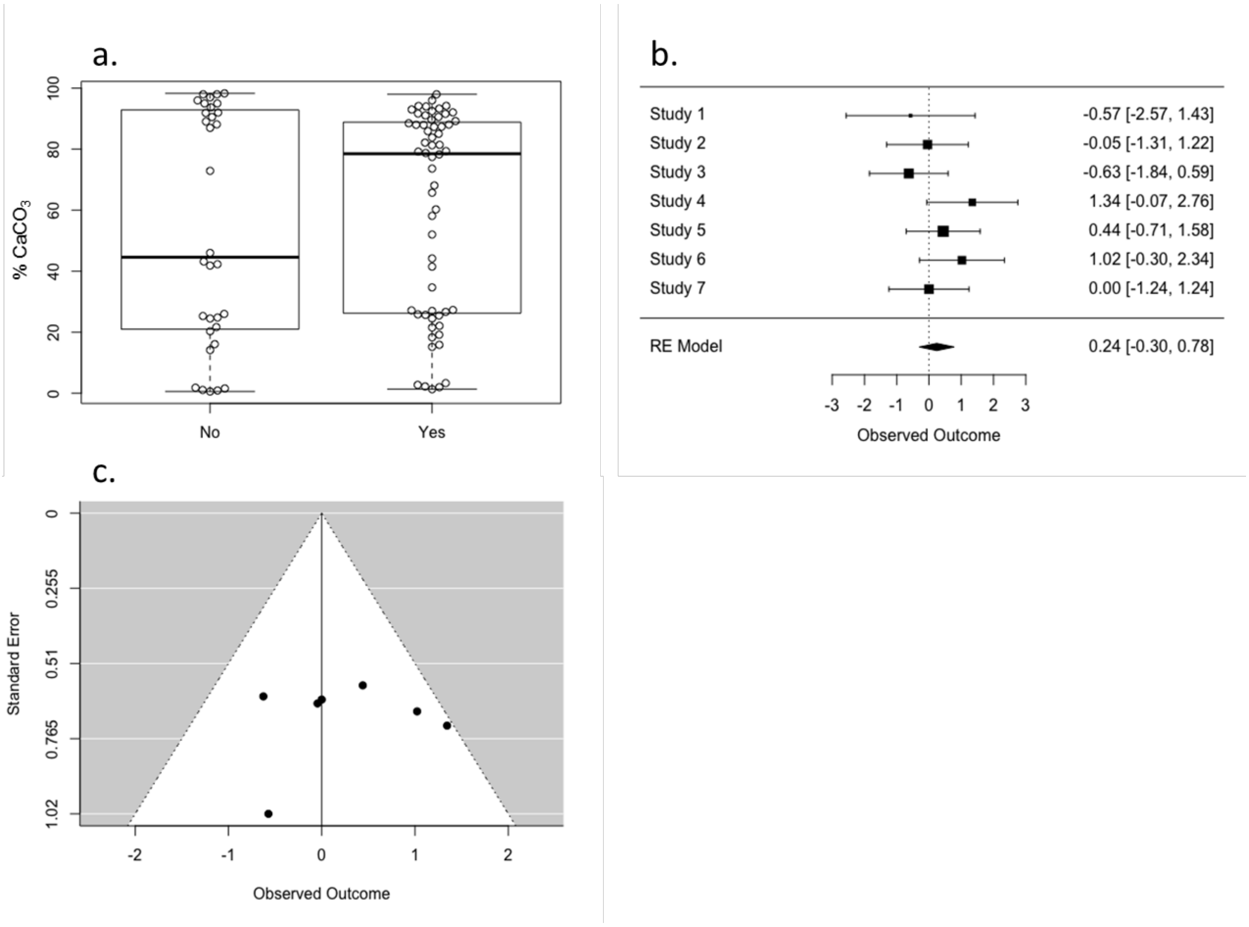

Supplementary Figure 2. Boxplot of raw data of the paired data (vegetated habitat, yes or no) used for the GLM (a). Boxplot shows each data point (circles) with the median (line through box) and the upper and lower quartiles (box limits), while the whiskers extend to the extreme data point but no more than 1.5 times the respective quartile. The forest plot of the meta-analysis of the paired data (b) The mean effect size and associated confidence intervals are

shown for each study as squares and lines and numbers on the right. The overall finding is shown at the bottom. The funnel plot of the meta-analysis (c). The funnel plot indicates potential sampling bias of meta-analysis by comparing the observed outcome (effect size) with the standard error and points outside of the white funnel indicate bias because of a high absolute value of the outcome and high standard error. See Methods for analysis details.

Supplementary Figure 3. Flow diagrams for: (a) the $CaCO_3$ – C_{inorg} burial rate data compilation in seagrass and mangrove. (b) the data compilation of $CaCO_3$ content (%DW) in seagrass.

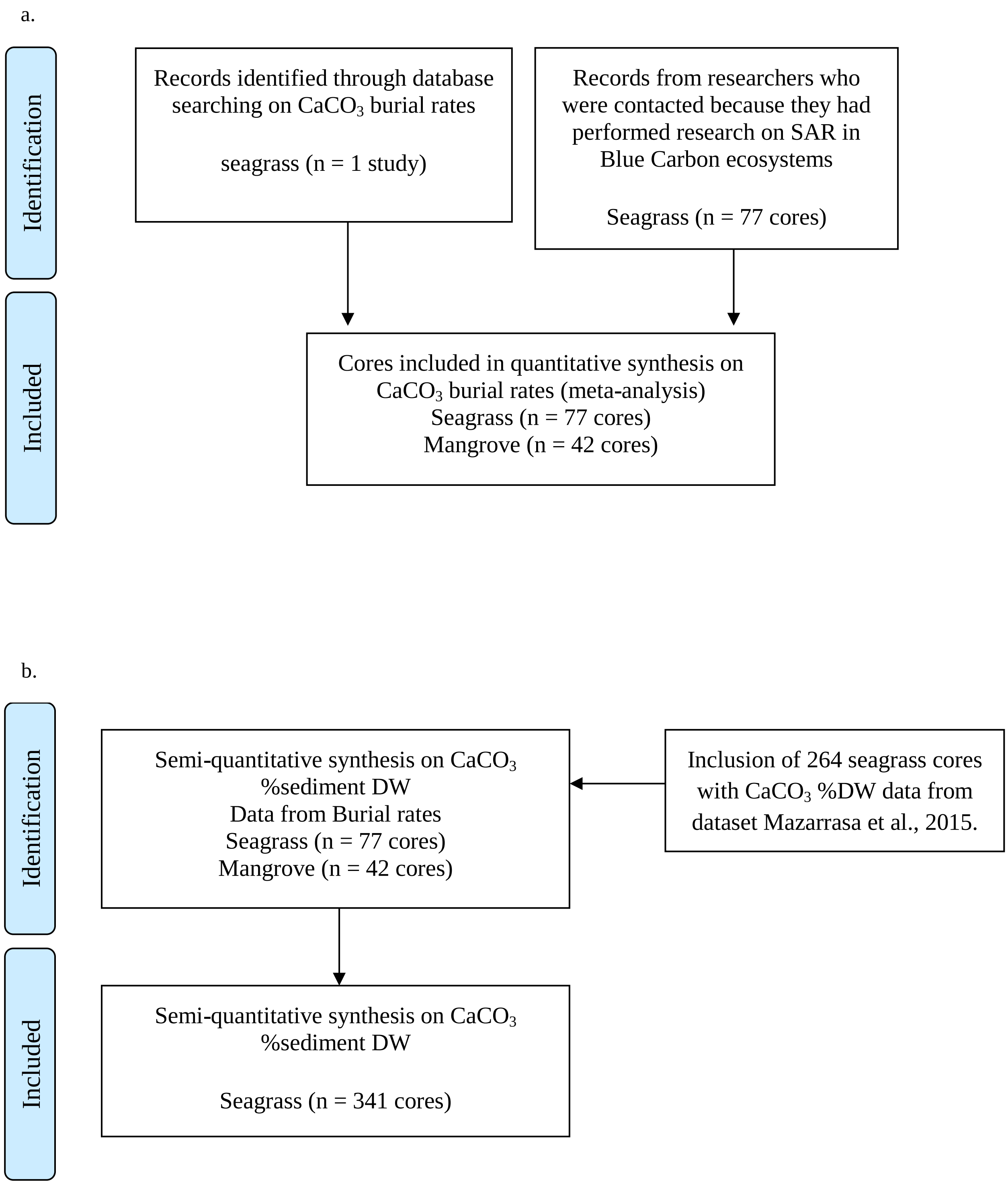

Supplementary Note 1: PRISMA 2009 Checklist according to **official template** from: Moher D, Liberati A, Tetzlaff J, Altman DG, The PRISMA Group (2009). Preferred Reporting Items for Systematic Reviews and Meta-Analyses: The PRISMA Statement. PLoS Med 6(7): e1000097. doi:10.1371/journal.pmed1000097. www.prisma-statement.org.

Section/topic	#	Checklist item
**TITLE**		
Title	1	Burial rate of $CaCO_3$ in seagrass and mangrove ecosystems and the influence of the proximity of external $CaCO_3$ sources on the $CaCO_3$ content in seagrass sediments – as part of the article "The role of carbonate burial in blue carbon ecosystems"
**ABSTRACT**		
Structured summary	2	Objectives: obtain a global value of burial rates of carbonates in seagrass and mangrove ecosystems and discuss this rate in relation to CO_2 emissions<br>data sources: author team and dataset from Mazarrasa et al., 2015.<br>study eligibility criteria: sediment cores from seagrass and mangrove sediment with C_{inorg} / $CaCO_3$ content (%DW) and sediment accumulation rate derived from ^{210}Pb methods.<br>Participants: V. Saderne, N. R. Geraldi, P. I. Macreadie, D. T. Maher, J. J. Middelburg, O. Serrano, H. Almahasheer, A. Arias-Ortiz, M. Cusack, B. D. Eyre, J. Fourqurean, H. Kennedy, D. Krause-Jensen, T. Kuwae, P. Lavery, C. E. Lovelock, N. Marba, P. Masqué, M. A. Mateo, I. Mazarrasa, K. J. McGlathery, M. P. J. Oreska, C. J. Sanders, I. R. Santos, J. M. Smoak, T. Tanaya, K. Watanabe and C. M. Duarte<br>synthesis methods; medians: general linear model (GLM)<br>results: burial rates of $CaCO_3$ in seagrass and mangrove ecosystems, as well as the effect of the presence / absence of coral reefs and / or lithogenic sources on the $CaCO_3$ content in seagrass and mangrove sediment.<br>limitations: geographical bias, lack of data<br>conclusions: There is significant burial of $CaCO_3$ in seagrass and mangrove ecosystem, but it cannot be related to the calcification / dissolution balance.<br>implications of key findings: need to investigate net calcification rates to determine CO_2 emission to the atmosphere due to calcification / dissolution in blue carbon ecosystems.<br>systematic review registration number: NA
**INTRODUCTION**		
Rationale	3	Mangrove forests and seagrass meadows have the capacity to elevate the seabed through the accretion of inorganic and organic particles[1] at global rates of ~0.5 cm yr^{-1} and ~0.2 cm yr^{-1}, respectively[1]. Sediment accretion in mangrove forests and seagrass meadows leads to the sequestration of organic carbon (C_{org})[2,3] originating from inside and outside of the vegetated ecosystem[4]. Although mangroves and seagrass ecosystems only occupy a limited fraction of the total coastal area (< 2%), they contribute 10% and 25% to the yearly C_{org} sequestration in the coastal zone[1,5]. Recognition of mangrove and seagrass meadows, together with saltmarshes, as sites of intense C_{org} burial led to the formulation of "Blue Carbon" strategies to mitigate and adapt to climate change, through conservation and restoration of these ecosystems[1,6,7,8]. The focus on Blue Carbon has provided substantial impetus to assess sediment C_{org} concentrations and burial rates in vegetated coastal ecosystems, which recently have been widely reviewed[9].<br>C_{org} generally represents a minor fraction (2-3%) of buried material within mangrove and seagrass sediments[10,11] (although this is highly variable[12]), the rest being siliciclastic and carbonate particles. A global assessment of the concentration of inorganic carbon concluded that C_{inorg} can exceed C_{org} concentration in seagrass sediments[13]. Seagrass and

		mangrove plants do not calcify *per se*; however, they provide habitats for an abundant associated calcifying fauna and flora (e.g. crabs, sea stars, snails, bivalves, calcified algae, foraminifera), whose shells and skeletons may be deposited and buried in the sediment along with the plant litter and the organic and inorganic particles imported from adjacent ecosystems.<br><br>Counterintuitively, $CaCO_3$ production represents a significant source of CO_2 to the atmosphere, since calcification produces CO_2 with a ratio of approximately 0.6 mol of CO_2 emitted per mol of $CaCO_3$ precipitated[14]. This has led to the argument that large $CaCO_3$ burial may partially offset CO_2 sequestration associated with C_{org} burial in some seagrass meadows and mangrove forests[15]. However, there are a number of caveats that affect these arguments and render inferences on the role of Blue Carbon ecosystems as net CO_2 sinks or sources based on the comparison of C_{org} and C_{inorg} sediment burial rates inconclusive[13,16]. First, only very few articles report the burial rates of $CaCO_3$ in mangrove and seagrass ecosystems[15,16,17], and the role of $CaCO_3$ burial in sediments and CO_2 emissions depends on the balance between dissolution and production. If $CaCO_3$ dissolution equals local calcification, then the burial of $CaCO_3$ is supported exclusively by allochthonous inputs and is neutral relative to CO_2 emissions or sequestration. If dissolution exceeds local calcification then $CaCO_3$ dynamics add to the CO_2 sink capacity of Blue Carbon ecosystems, even if $CaCO_3$, which must be subsidised from allochthonous sources, is buried in the sediments. Only if $CaCO_3$ dissolution is lower than local calcification does $CaCO_3$ burial result in CO_2 emissions.
Objectives	4	Here we address the current gap in global estimates of C_{inorg} burial in seagrass and mangrove ecosystems by providing first estimates of contemporary (last century) C_{inorg} burial rates. We rely on a global compilation of data on sediment chronologies (i.e. including radiometric dating of sediment cores with ^{210}Pb) and C_{inorg} concentrations from around the world. We then address the role of $CaCO_3$ burial in CO_2 emissions by resolving the source of the $CaCO_3$ buried in seagrass meadows (i.e. allochthonous or autochthonous).
**METHODS**		
Protocol and registration	5	There was no existing review protocol or registration information available.
Eligibility criteria	6	Eligible publications are articles providing a sediment core geochronology established with ^{210}Pb methods and either the $CaCO_3$ or inorganic carbon content of the sediment core (in %DW or g m^{-2} $sediment^{-1}$) in a seagrass or mangrove ecosystem.
Information sources	7	The dataset from Mazarrasa, Inés, Núria Marbà, Catherine E. Lovelock, Oscar Serrano, Paul S. Lavery, James W. Fourqurean, Hilary Kennedy et al. "Seagrass meadows as a globally significant carbonate reservoir." *Biogeosciences* (2015). Accessed in 2016.<br><br>Google scholar search for articles providing burial rates of $CaCO_3$ in seagrass and mangrove. Date of last search 2017.<br><br>data from co-authors team, published or not since the date of last search. (see item #9)
Search	8	Search of article with burial rates of $CaCO_3$ / C_{inorg} in seagrass and mangrove ecosystems on google scholar.<br><br>“seagrass” AND “210Pb” AND “CaCO3”; “seagrass”;; “seagrass” AND “210Pb” AND “inorganic carbon”.<br><br>“mangrove” AND “210Pb” AND “CaCO3”; “mangrove” AND “210Pb” AND “inorganic carbon”.
Study selection	9	Only studies with both sediment geochronology and $CaCO_3$ or C_{inorg} content in seagrass or mangrove were kept.<br><br>Only 1 article satisfying the criteria was found.<br><br>We therefore had to conduct additional searches.<br><br>We contacted a list of known experts in blue carbon science, having published one or several articles including sediment accretion rates in seagrass and mangrove ecosystems,

		and asked for unpublished data of $CaCO_3$ or C_{inorg} content in dated cores.
Data collection process	10	The study's authors sent us their raw datasets (detailed sediment core data).
Data items	11	All dated sediment cores slices with ^{210}Pb dating, allowing for a normalisation of the geochronology calculations and all sediment slices with $CaCO_3$ / C_{inorg} content.
Risk of bias in individual studies	12	Potential outliers were identified as any data points falling outside the 95% confidence interval of the fitted vs residual plot (using plot model function from the sjPlot package). The GLM analysis was then re-run, individually removing any study that had an outlier, to see if this study altered findings. For the traditional meta-analysis funnel plots were produced and an additional analysis was run after removing any study outside of the 95% confidence interval.
Summary measures	13	Mean %$CaCO_3$ and %C_{inorg} in every core. Recalculated ^{210}Pb sediment accumulation rates (SAR). C_{inorg} burial rates (g m^{-2} yr^{-1})
Synthesis of results	14	The SARs (cm yr^{-1}) from the literature were re-calculated according to the constant flux - constant sedimentation model[49] to have a coherent and comparable dating system between all cores.<br><br>The $CaCO_3$ concentration (% sediment dry weight) was calculated as the mean between all slices younger than 1900, for cores with the contemporary ^{210}Pb chronologies, and as the mean value between the surface and the deepest-dated slice for cores dated with ^{14}C. The C_{inorg} concentration in sediment (gC_{inorg} m^{-3}) was calculated from the dry bulk density (g m^{-3}) and the percentage of $CaCO_3$ content (using sediment dry weight), considering a mass ratio of 12% carbon in $CaCO_3$. The C_{inorg} burial rate (gC_{inorg} m^{-2} yr^{-1}) was then calculated as the product of the SAR and the C_{inorg} concentration for each sediment core. Cores with negligible content of $CaCO_3$ were also included in the calculation (see supplementary Fig. 1).<br><br>All cores from the same site or area and with similar presence or absence of allochthonous sources of $CaCO_3$ (see below) were treated as replicates for a global location and averaged for the analysis (geologic grouping). For seagrass, the 51 cores dated with ^{210}Pb were grouped into 17 locations, respectively (Fig. 2, 3). For mangroves, we compiled a total of 42 cores dated with ^{210}Pb in 8 locations (Fig. 2, 3). Seagrass locations ranged from tropical to sub-arctic locations, with 50 % of estimates derived from tropical and subtropical locations and 50 % from higher latitudes. Mangrove sediment derived mostly from subtropical locations (7 out of 8 locations), particularly in Australia and the Arabian Peninsula (supplementary Fig. 2).<br><br>We analysed the influence of the presence / absence of proximity of coral reefs and continental surface lithology (qualitative data), as potential allochthonous sources of $CaCO_3$ in seagrass and mangrove sediments (in % dry weight) (see dataset in supplementary dataset). For seagrass, we expanded our dataset by including $CaCO_3$ concentrations from 264 cores compiled by Mazarrasa et al. (2015)[13], reaching a total of 341 cores with measured $CaCO_3$ %DW.<br><br>We estimated the presence / absence of coral reefs using the map of the global distribution of warm-water coral reefs compiled by the UNEP-WCMC[50] and the presence – absence of nearby lithogenic sources using the global lithology map of Hartmann and Moosdorf (2012)[51] and the world soil map of the FAO/UNESCO[52]. The coring locations were associated with climate regions following the Köppen–Geiger classification system[53].<br><br>**Statistical analysis**<br><br>All data distributions were tested for normality to determine the most reliable central tendency measured with Shapiro-Wilks normality test (Statistica, Dell Software). None of the datasets of SAR, C_{inorg} concentration, C_{inorg} burial rate or $CaCO_3$ %DW were normally distributed (all $p < 0.05$). We therefore chose to use the median (IQR) as the most appropriate description of central tendency. Traditional meta-analysis tools, which

calculate effect sizes to standardize the difference between control and experimental treatments thereby allowing comparison among disparate response variables and weighting to account for unequal variance among studies, could not be used for this analysis for multiple reasons. These reasons include that the question posed and the studies available did not include experimental designs with paired control and experimental plots required for effect size calculations, that there was a single response variable facilitating direct comparison and data integration and, most importantly, that we used the raw data for each core. Instead, we ran a statistical test using a mixed effect general linear model (GLM) to determine the effect of coral reefs and lithogenic sources on the $CaCO_3$ %DW of the sediment. For sediments within seagrass meadows, the GLM included two fixed factors (presence / absence of coral reefs and of lithogenic sources), as well as the interaction between the two factors. For sediment within mangrove forests, the GLM included one fixed factor (presence / absence of allochthonous sources) because replication did not exist for all combinations of the two factors. The data had unequal samples among studies and studies were not evenly distributed around the globe (Fig. 1) which could result in pseudo-replication and biased results. To account for the data structure and minimize non-independence, we included 3 separate random variables, which included study, lithology grouping, and marine province. The marine province was determined for each sample location using the marine provinces of the world as defined by Spalding et al. (2007)[58]. Separate models where run for seagrass and mangrove sites. The statistical model was produced using the lmer function within the *lme4* package[59] and p-values were calculated with the *lmerTest* package[60]. The R^2 was calculated for the fixed and random effects using the r squared GLMM function in the *MuMIn* package[61]. The response variable was log transformed, which improved the model fit compared with raw data. The model fit was assessed by plotting the Q-Q plot (linear relationship) and the fitted values compared to the residuals (random distribution). To test if individual cores or studies were biased and having a disproportionate influence on findings, we systematically removed any studies that contained outlying samples as determined from being outside the 95% confidence interval for the fitted values vs. residuals comparison using the plot model function from *sjPlot* package[62]. This analysis was conducted in R version 3.4.2.

Reporting bias and its effect on findings is an important consideration for meta-analyses[63] and when the result from a meta-analysis was not the same as it would have been if data from all correctly conducted studies were included in the analysis[64]. A main cause of reporting bias is not publishing research because of a lack of merit as determined by the researcher, reviewer or editor[64]. As indicated by the data inclusion flow diagram (supplementary Figure 3), researchers often measured but did not publish data on soil calcium carbon content and authors needed to be directly contacted for these results. In addition, the researchers not only provided information from published studies but also unpublished data on calcium carbonate content (10 of 51 seagrass studies included in the analysis were not published). For these reasons, it is unlikely that our findings were affected by reporting bias. A subset of data collected for this study included the appropriate information to run both a GLM and a traditional meta-analysis (effect size could be calculated between paired data). The data included information from nine studies that measured $CaCO_3$ contend of sediment from both vegetated and unvegetated habitats. There were 92 cores samples with 32 from unvegetated and 60 from vegetated habitats (Supplementary Figure 2. A.). The GLM followed the same procedures as detailed in the main text except it had only two random factors, study and marine province, because study and lithology grouping differed in only one instance. For the meta-analysis, the data was paired for each study and the mean $CaCO_3$ %DW, number of samples, and standard deviation was calculated for vegetated and unvegetated cores for each study. Two studies included in the GLM were removed for the meta-analysis because they only had one core for unvegetated habitat and standard deviation could not be calculated, leaving seven comparisons for this analysis. Hedges' g was calculated for the effect size following Borenstein et al. (2009)[65] and a variance for each effect size was also calculated (V_g)[64], as indicated by equations:

		$$\text{Hedges'}g = \frac{(X_E - X_C)\,J}{SD_{pooled}}$$ $$SD_{pooled} = \sqrt{\frac{(n_E - 1)(SD_E)^2 + (n_C - 1)(SD_C)^2}{n_E + n_C - 2}}$$ $$J = 1 - \frac{3}{4\,(n_E + n_C - 2) - 1}$$ $$Vg = \frac{n_E + n_C}{n_E \times n_C} + \frac{g^2}{2\,(n_E + n_C)}$$ X_E and X_C are the mean (*n* is sample size) of vegetated and unvegetated sediments, along with SD_{pooled} and *J* which accounts for biases associated with different sample sizes. The meta-analysis included the same two random variables as the GLM and was conducted using the rma.mv function from the *metafor* package[66].
Risk of bias across studies	15	Missing some unpublished results from not contacted or non-responsive authors.
Additional analyses	16	A GLM was used because we had raw data and did not have paired control and experimental data as is usually used in traditional meta-analysis. However, we did have an overlapping data set that had both raw data and paired treatments which we analysed by GLM and traditional meta-analysis
Study selection	17	See supplementary Figure 2.
Study characteristics	18	Mean %$CaCO_3$ and %C_{inorg} in every core. Recalculated ^{210}Pb SAR. C_{inorg} burial rates (g m^{-2} yr^{-1}) (see supplementary table)
Risk of bias within studies	19	There were five studies that contained outliers. When separate GLM analyses were run excluding each of these studies individually the results of the tests were almost identical (p values remained the same order of magnitude). For the traditional meta-analysis, funnel plots indicated no biases and no individual study had significant differences as indicated by a forest plot
Results of individual studies	20	For the traditional meta-analysis, no individual study had significant differences as indicated by a forest plot. For the GLM no individual study with outliners had a strong influence on the results. In addition, the random factor of study included in the GLM should minimize any bias of individual studies and is indicated by the amount of variance attributed to this random variable. There was one exception for an individual study having an outlier which when removed changed the results. For the results from the mangrove data, one study from Florida had 20 cores, one of which was an outlier. When this study was removed from the analysis the effect of calcium carbonate source because significant. This is discussed in the study.
Synthesis of results	21	$CaCO_3$ support an important part of the sediment accretion rates (SAR) in seagrass ecosystems. Indeed, in 40 % of global locations, the $CaCO_3$ concentration was under 10 % dry weight (DW) while in 28 % of locations, the $CaCO_3$ content exceeded 80 %DW (see supplementary Figure1a). Overall, the median (interquartile range: IQR) global concentration of $CaCO_3$ in seagrass meadow sediments was 61 (56) % DW (mean ± SE of 54 ± 7).<br>In mangrove forests, we observe a large difference between the mean (± SE) and the median (IQR) $CaCO_3$ concentration: 3 (31) % and 21 ± 11 %. This is explained by strong bimodal distribution between the eight study locations examined, with a group of five locations with less than 5 %DW $CaCO_3$ in their sediments and three locations with $CaCO_3$ contents between 20 and 75 %DW (Shapiro-Wilks test, $p < 0.001$, see supplementary Figure1b). Converted into C_{inorg} concentration (after correction for the sediment bulk density), we obtain median (IQR) C_{inorg} concentrations in seagrass and mangrove sediments of 59 (66) and 1 (21) mgC_{inorg} cm^{-3} respectively (means ± SE of 63 ± 11 and 35 ± 17 mgC_{inorg} cm^{-3}) (Figure 2a).

		Using the median contemporary (last century – ^{210}Pb) SARs in seagrass and mangrove ecosystems compiled in this study (0.22 cm yr^{-1} and 0.23 cm yr^{-1}, respectively; Figure 2b), we estimate median (IQR) C_{inorg} burial rates in seagrass and mangrove ecosystems of 87 (154) and 6 (207) gC_{inorg} m^{-2} yr^{-1}, respectively (means ± SE of 182 ± 94 and 90 ± 43 gC_{inorg} m^{-2} yr^{-1}) (Figure 2c, Figure 3). These values correspond to vertical accretion rates of $CaCO_3$ of the order of 0.1 and 0.001 cm yr^{-1} in seagrass and mangrove ecosystems, respectively.<br><br>Our new estimates of contemporary burial rates are lower than the previous, indirect median estimate of C_{inorg} burial rate of 108 gC_{inorg} m^{-2} yr^{-1} (mean ± SE of 126 ± 31 gC_{inorg} m^{-2} yr^{-1}) by Mazarrasa et al. (2015)[13]. Our mean SAR values agree with previously reported global values[1,3].<br><br>Seagrass and mangrove ecosystems without potentially large adjacent allochthonous $CaCO_3$ sources have a remarkably lower median (IQR) sediment $CaCO_3$ content of 4 (15) and 1 (1) %DW (means ± SE of 11 ± 4 and 1.7 ± 0.8 %DW), respectively, than that of 59 (51) and 61 (27) %DW (means ± SE of 56 ± 5 and 53 ± 16 %DW) when at least one allochthonous $CaCO_3$ source was present (Figure 3).For sediments in seagrass meadows, the presence of coral reefs (t-value=4.68, df=48.5, $p < 0.0001$) and lithogenic sources (t-value = 4.76, df = 57.3, $p < 0.0001$) increased the amount of $CaCO_3$ in the sediment. There was a significant interaction between these factors (t-value = -3.29, df = 53.2, p = 0.0018) because the $CaCO_3$ %DW in presence of both allochthonous sources was less than would be expected than if these variables were additive. The statistical model explained 90 % of the variation in $CaCO_3$ %DW and the fixed factors accounted for 36 % of the variation, while the random variables accounted for 54 %. Mangrove sediment samples had a similar pattern to the seagrass meadows, and the presence of allochthonous sources had a marginally significant, positive effect on the amount of calcium carbonate in the sediment (t-value = 4.29, df = 1.81, p = 0.0596). The statistical model explained 91 % of the variation in sediment calcium carbonate and the fixed factors accounted for 71 % of the variation, while the random variables accounted for 20 % of the variation. In testing for biases of outlying cores and studies, we found that one study had an outlying data point and disproportionality influenced the results. The study from Western Florida had relatively low $CaCO_3$ but had an allochthonous source of $CaCO_3$. When this study was removed from the analysis, the presence of N allochthonous source became significant (t-value = 7.92, df = 4.16, p = 0.0012). This highlights the need for more studies on mangroves to determine the global influence of allochthonous sources on $CaCO_3$ content.<br><br>A comparison of paired, vegetated and unvegetated, sediment $CaCO_3$ %DW showed that vegetated and adjacent unvegetated sediments have similar carbonate concentrations, both using standard parametric statistics (GLM, t-value = 1.32, df = 83.1, p = 0.191) and meta-analysis (z-value=0.88, p = 0.379; Supplement Figure 2. A. B.), which also showed no evidence for reporting bias (all points within the 95 % confidence lines of the funnel plot, Supplement Figure 2. C). This provides further support to the notion that much of the carbonate buried in vegetated coastal sediments derives from allochthonous sources rather than being produced within the habitat.
Risk of bias across studies	22	There were five studies that contained outliers. When separate GLM analyses were run excluding each of these studies individually the results of the tests were almost identical (p values remained the same order of magnitude). For the traditional meta-analysis, funnel plots indicated no biases and no individual study had significant differences as indicated by a forest plot
Additional analysis	23	As mentioned, this study used both GLM and traditional analysis. Results were also discussed for basic description of findings and the related distributions.
**DISCUSSION**		
Summary of evidence	24	seagrass and mangrove ecosystems are places of intense carbonate burial<br><br>An important part of this burial seems to be of allochthonous origin (coral reef and or karstic origin)

Limitations	25	Very few data are available, especially in mangrove ecosystems. There is an important geographical bias. Given that the response variable was not reported in studies and that relevant researchers were contacted for both published and unpublished data, it was unlikely that this analysis was biased by reporting bias. In addition, data that did have the appropriate information for a traditional meta-analysis with effect sizes had no indication of reporting bias.
Conclusions	26	Burial rates of inorganic carbon cannot be used for the derivation of net CO_2 emission due to the balance between calcification and dissolution in seagrass and mangrove ecosystems.
**FUNDING**		
Funding	27	This research was supported by King Abdullah University of Science and Technology (KAUST) through baseline funding and workshop funding to C.M.D. Support from the Australian Research Council through grants LE170100219, DE160100443, DE170101524, DP150103286 and LE140100083 is acknowledged. D.K.-J. received financial support from the COCOA project under the BONUS programme, funded by the EU 7th Framework Programme and the Danish Research Council and her input is also a contribution to the CARMA project funded by the Independent Research Fund Denmark. AAO and PM acknowledge the support by the Generalitat de Catalunya (grant 2017 SGR-1588). This work is contributing to the ICTA `Unit of Excellence' (MinECo, MDM2015-0552). TK, KW, and TT were supported by JSPS KAKENHI (18H04156) and the Environment Research and Technology Development Fund (S-14) of the Ministry of the Environment, Japan. JMS was supported by the National Science Foundation under South Florida Water, Sustainability & Climate grant EAR-1204079.
