# Supplementary material for: Role of carbonate burial in Blue Carbon budgets
Source: Nat Commun. 2019 Mar 7;10:1106. doi: 10.1038/s41467-019-08842-6 (PMC6405941; doi:10.1038/s41467-019-08842-6)
Supplement: Supplementary file 3 — Description of Additional Supplementary Files [file 41467_2019_8842_MOESM3_ESM.pdf]

Description of Additional Supplementary Files

File Name: Supplementary Data 1

Description: A summary of the data used for the analysis.
